# Malaria knowledge and bed net use in three transmission settings in southern Africa

**DOI:** 10.1186/s12936-018-2178-8

**Published:** 2018-01-19

**Authors:** Mufaro Kanyangarara, Harry Hamapumbu, Edmore Mamini, James Lupiya, Jennifer C. Stevenson, Sungano Mharakurwa, Mike Chaponda, Philip E. Thuma, Lovemore Gwanzura, Shungu Munyati, Modest Mulenga, Douglas E. Norris, William J. Moss, For the Southern Africa International Centers of Excellence for Malaria Research

**Affiliations:** 1 Department of International Health, Johns Hopkins Bloomberg School of Public Health, Baltimore, MD USA; 2 Macha Research Trust, Macha, Choma District, Zambia; 3 Biomedical Research and Training Institute, Harare, Zimbabwe; 4 Tropical Disease Research Centre, Ndola Central Hospital, Ndola, Zambia; 5 Department of Molecular Microbiology and Immunology, Johns Hopkins Bloomberg School of Public Health, Baltimore, MD USA; 6 Department of Medical Laboratory Sciences, University of Zimbabwe College of Health Sciences, Harare, Zimbabwe; 7 Department of Epidemiology, Johns Hopkins Bloomberg School of Public Health, Baltimore, MD USA

**Keywords:** Insecticide-treated nets, Local knowledge, Malaria prevention and control, Zambia, Zimbabwe

## Abstract

**Background:**

Insecticide-treated nets (ITNs) reduce malaria morbidity and mortality in endemic areas. Despite increasing availability, the use of ITNs remains limited in some settings. Poor malaria knowledge is a barrier to the widespread use of ITNs. The goal of this study was to assess the levels of malaria knowledge and evaluate factors associated with bed net use among individuals residing in three regions of southern Africa with different levels of malaria transmission and control.

**Methods:**

A cross-sectional study was conducted on a sample of 7535 residents recruited from 2066 households in Mutasa District, Zimbabwe (seasonal malaria transmission), Choma District, Zambia (low transmission) and Nchelenge District, Zambia (high transmission), between March 2012 and March 2017. A standardized questionnaire was used to collect data on demographics, malaria-related knowledge and use of preventive measures. Multivariate logistic regression analyses were used to assess determinants of bed net use.

**Results:**

Most of the 3836 adult participants correctly linked mosquito bites to malaria (85.0%), mentioned at least one malaria symptom (95.5%) and knew of the benefit of sleeping under an ITN. Bed net ownership and use were highest in Choma and Nchelenge Districts and lowest in Mutasa District. In multivariate analyses, knowledge of ITNs was associated with a 30–40% increased likelihood of bed net use after adjusting for potential confounders across all sites. Other factors significantly associated with bed net use were age, household size and socioeconomic status, although the direction, strength and size of association varied by study site. Importantly, participants aged 5–14 years had reduced odds of sleeping under a bed net compared to children younger than 5 years.

**Conclusion:**

Relevant knowledge of ITNs translated into the expected preventive behaviour of sleeping under a bed net, underscoring the need for continued health messaging on malaria prevention. The implementation and delivery of malaria control and elimination interventions needs to consider socioeconomic equity gaps, and target school-age children to ensure access to and improve utilization of ITNs.

## Background

Globally, immense efforts have been made to control malaria, with the goal to ultimately eliminate malaria transmission [1]. Insecticide-treated nets (ITNs) are an important component of malaria control and elimination strategies. ITNs have been shown to reduce malaria episodes by 50% and under-five mortality by 17% [2]. Several studies from sub-Saharan Africa have also demonstrated community-wide benefits of ITNs on malaria-related morbidity and mortality [3, 4]. Despite individual and community-wide benefits, ITN use remains below universal coverage. A significant determinant of ITN use is ITN ownership [5]. The increased access to ITNs but lagging ITN use underscores the role of human behaviour in malaria transmission, treatment and control [6]. Numerous individual, household and community factors have been identified as determinants of ITN possession and use, including age, gender, level of education, socioeconomic status, household size, use of other preventive methods, and malaria-related knowledge [7–10]. Malaria knowledge is an important factor in the design and implementation of malaria control programmes. Several studies assessing the distribution of malaria knowledge in sub-Saharan Africa demonstrated inconsistent levels of malaria knowledge and indicated that misconceptions concerning the etiology and prevention of malaria still exist [11–18]. According to existing theories of health behaviour change, high levels of knowledge about the causation, transmission, prevention and treatment of malaria may facilitate changes in attitude, resulting in the adoption of positive preventive practices that reduce the risk of exposure to malaria and contribute to decreased malaria transmission [19].

The specific contribution of malaria knowledge to the adoption of malaria preventive behaviours is complex, and the strength and magnitude of reported associations has varied widely by context. Greater understanding of the level of malaria knowledge and association with malaria preventive behaviours in different transmission settings is essential for the implementation of evidence-based strategies to accelerate progress towards malaria elimination. The objectives of this study were to assess the underlying levels of malaria knowledge and evaluate the independent influence of malaria knowledge on bed net use in three settings in southern Africa with varying levels of malaria transmission and control. Findings will inform the development and targeting of context specific strategies to support and strengthen ongoing programmes to reduce malaria-related mortality and morbidity in southern Africa.

## Methods

### Study sites

**Fig. 1:**
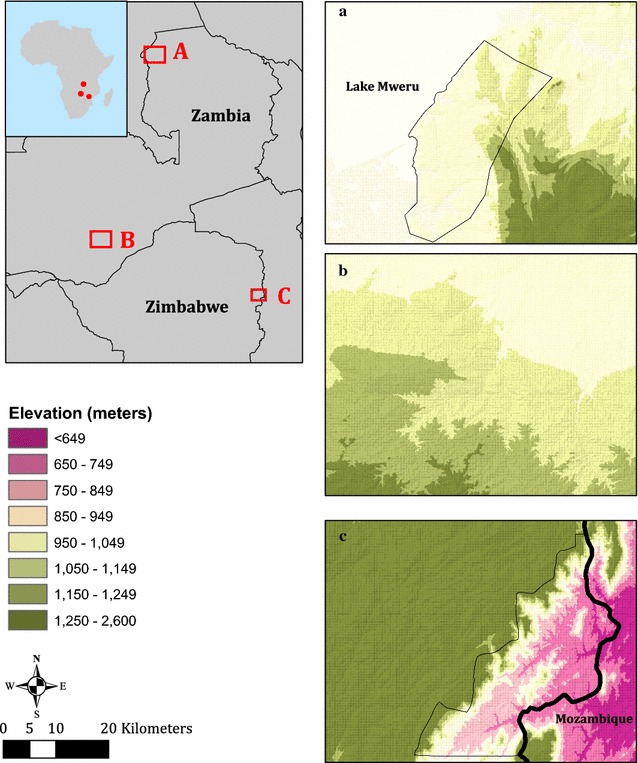
Location of Southern Africa International Centers of Excellence for Malaria Research study sites: **a** Nchelenge District, Zambia. **b** Choma District, Zambia. **c** Mutasa District, Zimbabwe

The study was based on a sample of 7535 participants representing 2066 randomly selected households from Mutasa District in eastern Zimbabwe, Choma District in southern Zambia and Nchelenge District in northern Zambia. The data used were acquired under the auspices of the Southern and Central Africa International Centers of Excellence for Malaria Research (ICEMR) project. The three study sites were specifically chosen by the Southern and Central Africa ICEMR to highlight variability in the epidemiology and transmission of malaria across southern Africa (Fig. 1, Table 1). In Choma District, malaria transmission is seasonal and the prevalence of malaria is low. By contrast, in Nchelenge District, which lies along Lake Mweru and borders the Democratic Republic of Congo, malaria transmission is intense with little or no seasonal fluctuations. Malaria transmission in Mutasa District is highly seasonal, with malaria-related morbidity and mortality peaking during the rainy season.

**Table 1:** Epidemiological characteristics of malaria in the Southern Africa International Centers of Excellence for Malaria Research (ICEMR) study sites

	Choma District	Nchelenge District	Mutasa District
Location	Southern Province, Zambia	Luapula Province, Zambia	Manicaland Province, Zimbabwe
Geographical position	16° 23.583′S, 26° 47.433′E	9° 19.115′S, 28° 45.070′E	18° 23.161′S, 32° 59.946′E
Average elevation above sea level (m)	1100	807	912
Seasons	Rainy: November–April Cool dry: May–July Hot dry: August–October	Rainy: November–April Cool dry: May–August Hot dry: September–October	Rainy: November–April Cool dry: May–July Hot dry: August–October
Malaria transmission	Seasonal but low (pre-elimination)	Intense with little or no seasonal fluctuations	Seasonal, unstable and epidemic in nature; decreased over past 3 years following IRS
Primary mosquito vector	*Anopheles arabiensis*	*Anopheles funestus* and *Anopheles gambiae*	*Anopheles funestus*
Malaria control phase	Successful malaria control	Ineffective malaria control	Resurgent malaria after previous control; again decreasing
Current malaria control interventions	Insecticide-treated bed nets and reactive screen and treat. Indoor residual spraying and mass drug administration at a limited scale	Insecticide-treated bed nets, indoor residual spraying and case management with artemisinin-based combination therapy	Insecticide-treated bed nets, indoor residual spraying and case management with artemisinin-based combination therapy
Estimated population	205,000	148,000	180,000
Main economic activity	Cattle herding and subsistence farming	Subsistence farming and fishing	Subsistence and commercial farming

**Fig. 2:**
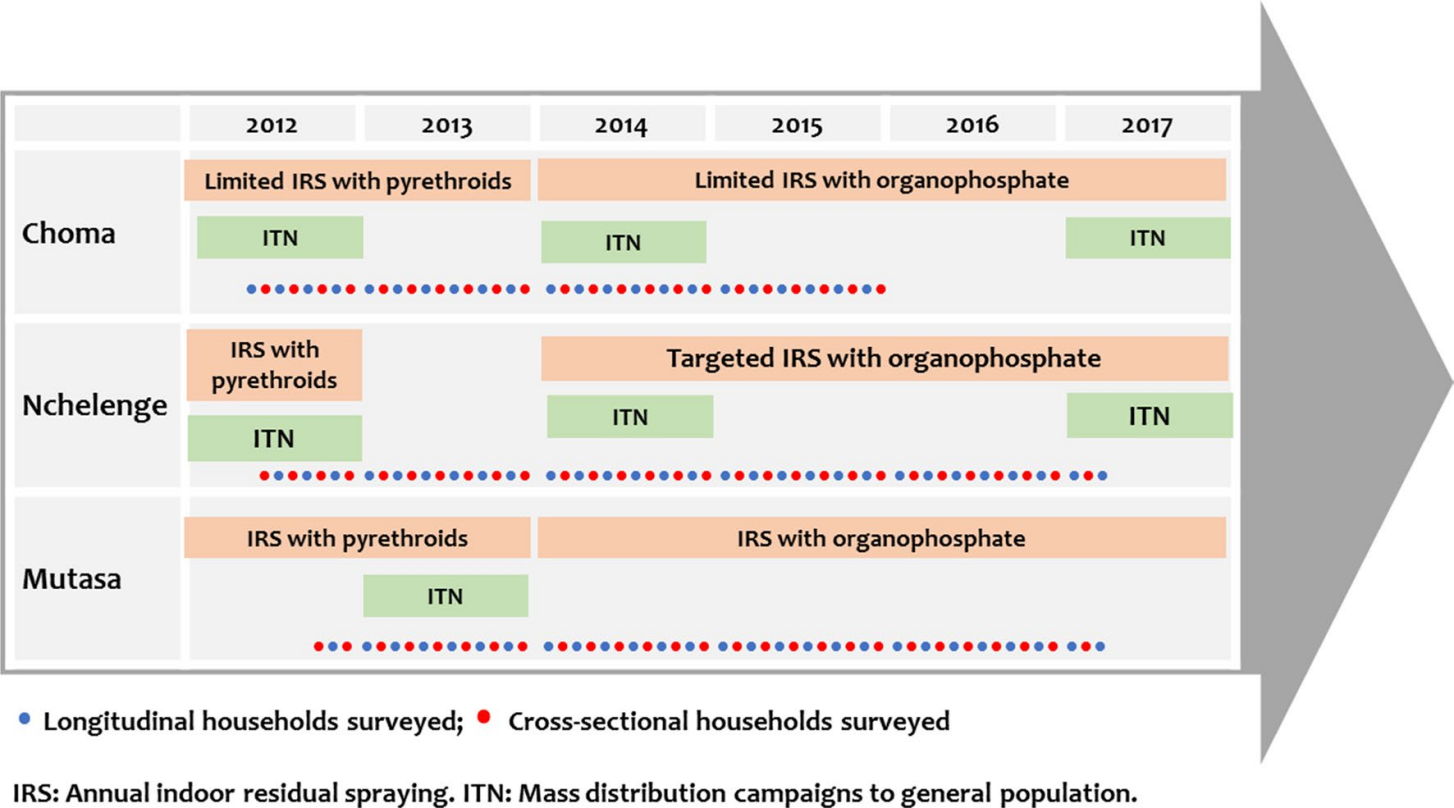
Study timeline

Several malaria control activities including the distribution of ITNs and application of indoor residual spraying (IRS) occurred during the study period (Fig. 2). In Choma District, mass distribution of free, long-lasting insecticidal nets (LLINs) occurred in 2007, 2012 and 2014 [7, 20]. In Nchelenge District, mass LLIN distribution campaigns took place in 2007, 2011 and 2014. Annual rounds of IRS began in 2006, first with pyrethroids, then carbamates [21]. Since 2014, the organophosphate pirimiphos-methyl has been used for targeted IRS in the study area. Similar to Nchelenge District, there was a programmatic switch from pyrethroid-based to organophosphate-based IRS in Mutasa District in 2014. Following the introduction of pirimiphos-methyl, Mutasa District has experienced moderate reductions in malaria incidence [22]. Universal ITN distribution to the general population was conducted in 2013, with distributions to vulnerable populations (e.g. school age children) in 2014 and 2015.

### Study design and procedures

The study design and procedures have been described elsewhere [23–25]. Briefly, high resolution satellite images of the study areas were used to establish sampling frames and study households were randomly selected for enrolment in the cross-sectional study arm or the prospective longitudinal cohort. Households enrolled in the longitudinal cohort were visited every other month, while households in the cross-sectional arm were surveyed only once during the study period. Study enrollment began in March 2012 in Choma District, April 2012 in Nchelenge District and October 2012 in Mutasa District (Fig. 2). Across all sites, cross-sectional surveys and longitudinal surveys were conducted during alternating months during the study period. Enrollment of new participants from randomly selected households in Choma District ended in December 2014; thereafter, participants have been recruited under the reactive test and treat programme [26]. Study enrollment is still ongoing in the other sites. For the purposes of this analysis, December 31, 2015 was set as the cutoff date for Choma District and March 31, 2017 for Nchelenge and Mutasa Districts. The analysis was restricted to data from all visits in the cross-sectional arm and the initial baseline visits for the longitudinal cohort.

Data were collected using standardized data collection instruments that were field tested to ensure reliability and validity. In all selected households, the head or another responsible adult member of the household was interviewed after informed consent was given. A complete listing of all residents of selected households was obtained and all residents were eligible to participate regardless of age, gender or pregnancy status. The availability of household assets and characteristics of the house structure were recorded. An interviewer-administered questionnaire was used to gather data on malaria-specific knowledge for adult participants and presenting symptoms, health-seeking behaviours and malaria prevention practices for all participants. At the end of each interview, a blood sample was collected for a malaria rapid diagnostic test (RDT). Participants with positive test results were treated as per the country-specific malaria treatment guidelines.

### Measures

Several measures reflecting population coverage of bed nets were calculated as recommended by the Roll Back Malaria (RBM) partnership [5, 27], using data on direct observation of bed net ownership and self-reported bed net use. The term ‘bed net’ was used to encompass insecticide-treated and untreated bed nets, although long-lasting insecticidal nets (LLINs) are the standard in all study sites. The primary outcome measure was individual-level bed net use among those with living in a household with any bed net. The primary exposure of interest was knowledge of the benefit of sleeping under an ITN derived from responses to an unprompted open-ended question on the ways to prevent malaria. Demographic characteristics included gender and age of the respondent (< 5, 5–14, 15–34, and ≥ 35 years). Three variables, namely, educational level of the head of the household (none or primary, secondary education and tertiary education or higher), permanent employment status of the head of the household (employed or unemployed) and a wealth index, were used as proxies for socioeconomic status. The wealth index was calculated using principal components analysis based on ownership of assets (radio, television, refrigerator, bicycle, motorcycle, and car or truck) and house characteristics (source of drinking water, source of energy for cooking and floor material) [28]. The index was divided into three tertiles—‘poorest’, ‘less poor’ and ‘least poor’. Other variables controlled for in the analysis were based on prior literature and included number of children under 5 years, number of bed nets in household, and household size.

### Analysis

Frequency distributions were used to describe the sample population, quantify knowledge about the cause, symptoms and prevention measures for malaria, and describe bed net ownership and use. Pearson’s Chi squared test was used to compare sociodemographic and household characteristics across study sites. To determine if knowledge of ITNs as a preventive measure significantly increased the odds of bed net use, logistic regression analyses were performed separately for each study site. To account for within-household correlation, univariate and multivariate logistic regression analyses were conducted using generalized estimated equations (GEE) [29]. Age and gender were included in all multivariate models to control for potential confounding. Calendar year and season (rainy/dry) were also included to account for secular trends and seasonality in the outcomes. Adjusted odds ratios (aOR) with 95% confidence intervals (CI) were computed from the final GEE models. A p value of less than 0.05 was considered statistically significant. Analyses were performed using STATA 14.2 (College Station, Texas).

## Results

### Characteristics of the study population

The analysis included 7535 participants, with 1761 from Choma District, 3405 from Nchelenge District and 2369 from Mutasa District (Table 2). One in five participants was under 5 years of age (19.3%), and slightly more than half of participants were female (55.3%). There were some differences in sociodemographic characteristics by study site. Compared to the other study sites, participants in Choma District tended to reside in larger households, with 46.7% of participants residing in a household with 7 or more members (p < 0.001). Compared to Choma and Nchelenge Districts, a higher proportion of participants in Mutasa District belonged to households headed by individuals who had completed secondary or tertiary education (55.6%, p < 0.001) and were in permanent employment (46.5%, p < 0.001). Participants from Mutasa District were also relatively better off than their counterparts as a higher proportion of participants reported the use of electricity for cooking (7.5%, p < 0.001), piped water for drinking (28.0%, p < 0.001) and a finished floor in the home (88.4%, p < 0.001). A higher proportion of participants in Mutasa District lived in households that had been covered by IRS in the previous 6 months than the two Zambian sites (40.5%, p < 0.001).

**Table 2:** Study population characteristics by study site

Variables	Choma District N = 1761		Nchelenge District N = 3405		Mutasa District N = 2369		Total N = 7535		p value^a^
	n	%	n	%	n	%	n	%	
Age (years)									< 0.001
< 5	383	21.8	670	19.7	402	17.0	1455	19.3	
5–14	541	30.7	1022	30.0	533	22.5	2096	27.8	
15–34	432	24.5	955	28.1	742	31.3	2129	28.3	
≥ 35	405	23.0	758	22.3	692	29.2	1855	24.6	
Gender									0.21
Male	810	46.0	1530	44.9	1026	43.3	3366	44.7	
Female	951	54.0	1875	55.1	1343	56.7	4169	55.3	
Education level of head of household									< 0.001
Primary or less	990	56.2	2329	68.4	1051	44.4	4370	58.0	
Secondary	709	40.3	1025	30.1	1112	46.9	2846	37.8	
Tertiary	62	3.5	51	1.5	206	8.7	319	4.2	
Employment status of head of household									< 0.001
Employed	132	7.5	229	6.7	1102	46.5	1463	19.4	
Unemployed	1629	92.5	3172	93.3	1267	53.5	6068	80.6	
Household asset ownership									
Radio	1286	73.1	2190	64.3	1281	53.9	4757	63.1	< 0.001
Television	480	27.3	242	7.1	670	28.2	1392	18.5	< 0.001
Fridge	24	1.4	57	1.7	185	7.8	266	3.5	< 0.001
Bicycle	1353	76.9	2391	70.2	634	26.7	4378	58.1	< 0.001
Motorcycle	37	2.1	28	0.8	85	3.6	150	2.0	< 0.001
Car or truck	136	7.7	5	0.1	212	8.9	353	4.7	< 0.001
Source of drinking water: piped water	16	2.0	17	1.0	390	28.0	423	11.1	< 0.001
Source of energy for cooking: electricity	4	0.5	22	1.3	104	7.5	130	3.4	< 0.001
Main material of floor: finished flooring	225	28.5	209	12.9	1229	88.4	1662	43.7	< 0.001
Number of household members									< 0.001
1–2	93	5.3	596	17.5	454	19.2	1143	15.2	
3–6	846	48.0	2235	65.6	1259	53.1	4340	57.6	
≥ 7	822	46.7	574	16.9	656	27.7	2052	27.2	
Visited health facility for malaria in past 6 months	238	13.5	1906	56.0	687	28.9	2831	37.6	< 0.001
Visited health facility for malaria in past month	32	1.8	791	23.2	247	10.4	1070	14.2	< 0.001

^a^Chi squared test

### Malaria-related knowledge

Of the 3843 participants aged 16 years or older and eligible to respond to questions related to malaria knowledge, 3836 (99.9%) responded to the malaria knowledge questionnaire (Table 3). The majority (85.0%) of respondents linked malaria to a mosquito bite, with the highest proportion (89.4%) in Choma District, the setting with the lowest malaria burden. A few respondents associated malaria with dirty surroundings (3.9%), drinking bad water (3.6%), and other causes including eating bad food, fresh fruit, maize or sugar cane (4.1%). Among those who correctly linked malaria to a mosquito bite, 2.7% also cited one or more incorrect causes. The most frequent symptoms listed as presumptive for malaria varied by site. In Choma District, respondents most commonly associated malaria with headache (68.3%), chills (62.1%) and fever (47.4%). In Nchelenge District, chills (56.3%), fever (35.8%) and body ache or pain (33.0%) were the most commonly reported symptoms of malaria. By contrast, headaches (70.0%), weakness or fatigue (60.2%) and chills (50.5%) were the most commonly reported symptoms in Mutasa District. Overall, almost all respondents (95.5%) mentioned at least one common symptom of malaria (fever, chills, headache, weakness or fatigue, and body ache or pain), and 29.0% could mention three or more of the common symptoms of malaria. Sleeping under a mosquito net was the most commonly reported measure thought to prevent malaria (73.1%), with the highest level of knowledge of the benefits of net use in Choma District (87.3%) and the lowest in Mutasa District (67.2%). Seeking early treatment (11.5%), keeping surroundings clean (10.9%), burying mosquito breeding sites (8.34%) and indoor residual spraying (6.7%) were other preventive measures reported. A minority of respondents linked eating clean food to the prevention of malaria (3.7%). Information about malaria was commonly received from health workers in health facilities (54.4%), schools (15.7%), and the community (8.6%). Less frequently mentioned sources of information about malaria were radios, newspapers, posters, friends, relatives, non-governmental organizations and the study team.

**Table 3:** Reported knowledge on malaria causes, symptoms and preventive measures by study site

	Choma District N = 789		Nchelenge District N = 1643		Mutasa District N = 1404		Total N = 3836	
	n	%	n	%	n	%	n	%
Knowledge of causes of malaria								
Mosquito bites	705	89.4	1344	81.8	1212	86.3	3261	85.0
Also cited other cause(s)	48	6.8	21	1.6	19	1.6	88	2.7
Dirty surroundings	72	9.1	24	1.5	54	3.8	150	3.9
Drinking bad water	80	10.1	36	2.2	21	1.5	137	3.6
Other causes^a^	57	7.2	38	2.3	62	4.4	157	4.1
Knowledge of malaria symptoms								
Mentioned 3 or more common symptoms of malaria^b^	264	33.5	267	16.3	583	15.2	1114	29.0
Chills	490	62.1	925	56.3	708	50.5	2123	55.4
Headache	539	68.3	491	29.9	982	70.0	2012	52.5
Fever	374	47.4	588	35.8	476	33.9	1438	37.5
Weakness or fatigue	159	20.2	188	11.4	845	60.2	1192	31.1
Body ache or pain	157	19.9	542	33.0	209	14.9	908	23.7
Vomiting	243	30.8	94	5.7	499	35.6	836	21.8
Other symptoms^c^	374	47.4	248	15.1	569	40.5	1191	31.0
Knowledge of the prevention of malaria								
Sleep under a mosquito net	689	87.3	1173	71.4	943	67.2	2805	73.1
Seek early treatment	134	17.0	145	8.8	161	11.5	440	11.5
Keep surroundings clean	113	14.3	49	3.0	257	18.3	419	10.9
Bury mosquito breeding sites	84	10.6	36	2.2	203	14.5	323	8.4
Spray insecticide inside the house	23	2.9	24	1.5	211	15.0	258	6.7
Take medicine to prevent malaria	10	1.3	87	5.3	91	6.5	188	4.9
Eat clean food	80	10.1	34	2.1	27	1.9	141	3.7
Other measures^d^	10	1.3	34	2.1	180	12.8	224	5.8
Source of malaria knowledge								
Health care worker at clinic or hospital	539	68.3	754	45.9	793	56.5	2086	54.4
School	110	13.9	243	14.8	248	17.7	601	15.7
Community health worker	32	4.1	76	4.6	223	15.9	331	8.6
Other sources^e^	88	11.2	224	13.6	131	9.3	443	11.5

Percentage total exceed 100 because of multiple responses

^a^Other causes included breathing bad air, cold related and eating bad food, fresh fruits, maize or sugar cane

^b^Common symptoms of malaria were fever, chills, headache, weakness or fatigue, and body ache or pain

^c^Other symptoms included diarrhea, coughing, flu-like symptoms, yellow eyes or skin and thirst

^d^Other preventive measures included keeping the skin covered, wearing insect repellent, having screens on the windows, burning mosquito coils, burning a fire in the house and not going outside at certain times

^e^Other sources were radio, newspapers, posters in health post or health center, friends or relatives, non-governmental organizations and the ICEMR study team

### Bed net ownership, access and use

Bed net ownership, access and use varied by study site, with Mutasa District reporting the lowest levels. At the household level, ownership of any bed net was 69.9%, while ownership of sufficient bed nets (i.e. at least one bed net for every two members) was 39.7% (Table 4). At the population level, access to a bed net within the household was 39.2%, while bed net use was 31.8%. The proportion of the population using bed nets was fairly similar to the proportion of the population with access to a bed net, indicating an average of two users per net. Unavailability of bed nets (50%) and the perceived lack of mosquitoes (26.5%) were the main reasons reported by households for not owning a net, while the perceived lack of mosquitoes (17.4%) and heat (10.1%) were the main reasons for not sleeping under a bed net. By contrast, in the low transmission setting of Choma District, 78.2% of household owned any bed net and 70.8% of the population reported sleeping under a bed net. Indicators of bed net ownership, access and use for Nchelenge District did not vary appreciably from Choma District despite the higher malaria transmission intensity. In both Zambian sites, cost and lack of knowledge of where to obtain a bed net were the main barriers to bed net ownership reported (Choma: 32.1 and 22.5% respectively; Nchelenge: 26.7 and 21.6% respectively). However, the perceived lack of mosquitoes (5.2%) was the most cited reason for non-use of available bed nets in Choma District, while the most common reason in Nchelenge District was the state of the available net (old, dirty or in need of retreatment; 4.8%).

**Table 4:** Bed net ownership, access and use by study site

	Choma District	Nchelenge District	Mutasa District	Total
Population with access to an ITN within their household (%)	70.8	57.8	39.2	55.0
Population that slept under an ITN (%)	55.6	57.4	31.8	49.0
Children under 5 years old who slept under an ITN (%)	60.8	59.7	34.9	53.2
Households with at least one ITN (%)	78.2	77.8	69.9	75.3
Households with at least one ITN for every two people (%)	49.6	49.0	39.7	46.0
Households sprayed in the last 6 months (%)	2.3	14.5	42.9	21.9
Households with at least one ITN and/or sprayed by IRS in the last 6 months (%)	79.0	80.7	81.8	80.8
Households with at least one ITN for every two people and/or sprayed by IRS within the last 6 months (%)	51.6	55.9	64.7	58.1
Reasons for not owning a bed net at the household level^a^				
Nets not available	7.0	26.1	50.5	31.3
No mosquitoes	16.8	19.8	26.5	21.7
Too expensive	32.1	26.9	2.3	18.8
Don’t know where to get a bed net	22.5	21.6	0	13.7
Heat	3.5	0.6	3.4	2.2
Other reasons^a^	10.8	1.4	7.0	5.5
Reasons for not sleeping under an available bed net at the individual level^b^				
Heat	3.2	1.0	10.1	5.5
Net is old, dirty or needs to be retreated	0.4	4.8	2.7	3.0
Not enough bed nets	2.8	1.1	0.2	1.0
Does not protect against mosquitoes	3.9	0	0	0.8
Lack of mosquitoes	5.2	0.5	17.4	9.0
Unable to hang over sleeping space	0.6	0.9	2.9	1.7
Net is itchy	1.7	0.1	1.4	1.0
Other reasons^b^	0.6	0.9	0.3	0.6

Percentage total exceed 100 because of multiple responses

^a^Other reasons for not owning a bed net included lack of protection against mosquitoes, nets only for children and pregnant women, not the rainy or malaria season and sleeping space is outside or changes too often

^b^Other reasons for not sleeping under an available bed net included not the rainy or malaria season, keeping nets for children and pregnant women, sleeping space is outside, and frequent changes to sleeping place

### Factors associated with bed net use

In Choma District, multivariate analyses restricted to individuals residing in households with any bed nets demonstrated marginal evidence of a higher odds of bed net use among respondents with knowledge of ITNs as a preventive measure (aOR 1.40, 95% CI 0.97–2.03) (Table 5). Compared to individuals aged less than 5 years, the odds of bed net use were greater in the ≥ 35 years age group (aOR 2.38; 95% CI 1.55–3.67) and lesser in the 5–14 years age group (aOR 0.57; 95% CI 0.41–0.79). The odds of bed net use decreased with large household size (3–6 members: aOR 0.29; 95% CI 0.14–0.58; 7+ members: aOR 0.32; 95% CI 0.16–0.67 relative to one to two members). Also, residing in a household with three or more bed nets or with at least one child under 5 years increased the odds of bed net use (aOR 2.52; 95% CI 1.75–3.62; aOR 1.42; 95% CI 1.05–1.96, respectively).

**Table 5:** Factors associated with bed net use by study site

	Choma District N = 1446		Nchelenge District N = 2774		Mutasa District N = 1803	
	aOR (95% CI)	p	aOR (95% CI)	p	aOR (95% CI)	p
Age (years)						
< 5	Reference		Reference		Reference	
5–14	0.57 (0.41–0.79)	0.001	0.49 (0.38–0.62)	< 0.001	0.76 (0.54–1.06)	0.1
15–34	1.20 (0.81–1.78)	0.4	1.34 (0.89–2.00)	0.2	1.16 (0.76–1.78)	0.5
≥ 35	2.38 (1.55–3.67)	< 0.001	3.99 (2.57–6.20)	< 0.001	1.81 (1.18–2.79)	0.007
Female gender	1.05 (0.84–1.33)	0.7	1.34 (1.11–1.61)	0.001	0.89 (0.73–1.09)	0.3
Has knowledge of ITNs	1.40 (0.97–2.03)	0.07	1.35 (1.11–1.64)	0.003	1.27 (1.02–1.58)	0.03
Household wealth tertile						
Poorest	Reference		Reference		Reference	
Less poor	1.16 (0.87–1.56)	0.3	1.46 (1.20–1.78)	< 0.001	1.03 (0.77–1.40)	0.8
Least poor	1.06 (0.78–1.44)	0.7	1.56 (1.19–2.06)	0.001	0.74 (0.57–0.96)	0.02
Number of household members						
1–2	Reference		Reference		Reference	
3–6	0.29 (0.14–0.58)	0.001	0.35 (0.25–0.50)	< 0.001	0.74 (0.54–1.00)	0.05
≥ 7	0.32 (0.16–0.67)	0.002	0.25 (0.17–0.37)	< 0.001	0.65 (0.45–0.93)	0.02
At least one child under 5 years in household	1.43 (1.05–1.96)	0.03	1.70 (1.35–2.14)	< 0.001	1.26 (0.98–1.61)	0.07
Three or more bed nets in household	2.52 (1.75–3.62)	< 0.001	1.42 (0.99–2.04)	0.06	1.93 (1.37–2.72)	< 0.001

*aOR* adjusted odds ratio, *CI* confidence interval. Multivariate logistic regression model also included season and calendar year

Consistent with patterns observed among residents of Choma District, in Nchelenge District awareness of ITNs as a preventive measure was associated with statistically significant increased odds of bed net use (aOR 1.35; 95% CI 1.11–1.64). Associations with bed net use of similar magnitude and significance were observed for age, household size, the presence of at least one child under 5 years and household ownership of three or more bed nets. However, the odds of bed net use were significantly higher among females (aOR 1.34; 95% CI 1.11–1.61) and individuals from households of higher socio-economic status (least poor aOR 1.56; 95% CI 1.19–2.06).

Knowledge of ITNs was predictive of bed net use in Mutasa District (aOR 1.27; 95% CI 1.02–1.58). Age was associated with bed net use, with the odds of bed net use significantly higher among respondents 35 years or older (aOR 1.81; 95% CI 1.18–2.79). The odds of bed net use were reduced by 26% among individuals residing in the least poor households compared to the poorest households (aOR 0.74; 95% CI 0.57–0.96). The presence of at least three bed nets in the household increased the odds of bed net use by 93% (aOR 1.93; 95% CI 1.37–2.72).

## Discussion

This study assessed levels of malaria knowledge and factors associated with bed net use in three different transmission settings in Mutasa District, Zimbabwe, Choma District, Zambia and Nchelenge District, Zambia. In general, most respondents (85%) knew the cause of malaria, albeit 2.7% of those also cited an incorrect cause of malaria. Most respondents (73.1%) were aware of the protective benefit of sleeping under an ITN and could list at least one potential symptom of malaria (95.5%). Similar levels of knowledge of the cause, prevention and symptoms of malaria were recently reported in other geographic areas in Zambia and Zimbabwe [7, 11, 18, 30–32]. These findings, in conjunction with recent improvements in the coverage of ITNs, highlight the success of malaria prevention education delivered by facility-based and community-based health workers, who were identified as the main source of malaria messages. However, our study suggests that some misconceptions still prevail. In Choma District, while 9 in 10 participants linked malaria to mosquito bites, about 1 in 10 residents still believed that drinking bad water causes malaria and 1 in 5 believed that dirty surroundings contribute to malaria. One explanation is that in this as well as other settings, the local term for ‘malaria’ is often used to describe fever and general malaise [15, 33]. Misconceptions and misinformation have continued amid intensified efforts to control and eliminate malaria. Ownership of a radio was common, yet less than 1% of participants reported hearing health messaging on malaria prevention through these mediums, representing a critical missed opportunity for the wider dissemination of health messaging to stimulate changes in knowledge and positive health behaviour change.

Across all transmission settings, the proportion of households with at least one bed net ranged from 69.9 to 78.2%, but the proportion of households with at least one bed net per two members was substantially lower (range 39.7–49.6%), suggesting a considerable intra-household ownership gap. These findings are consistent with national estimates of household bed net ownership rates from recent national surveys in Zimbabwe (60.3%) and Zambia (79.5%) [34, 35]. Notably, the lower ownership rates in Zimbabwe compared to Zambia may reflect national policy in Zimbabwe aimed at achieving universal malaria protection by deploying either ITNs or IRS, but not both, to malarious areas. This explanation is supported by the present study’s finding that, while the proportion of households with any bed net was lower in Zimbabwe compared to the other sites, the proportion of households protected by bed nets or IRS or both was similar across the three sites.

In the present study, respondents who reported knowledge of the protective efficacy of ITNs had increased odds of sleeping under a bed net (up to 40%). Results from this large community-based cross-sectional study are in concordance with other studies that demonstrated malaria knowledge is strongly associated with preventive behaviours related to malaria in sub-Saharan Africa [18, 32]. Associations were also found between bed net use and socioeconomic status, albeit with divergent directions of associations. For instance, in Nchelenge District, participants residing in the ‘least poor’ households had a greater likelihood of bed net use, compared to their counterparts of similar characteristics in the ‘poorest’ households. This finding was supported by the observation that the most cited reason for not owning a bed net in Nchelenge District was affordability. By contrast, in Mutasa District, increased household wealth was associated with a decreased odds of bed net use. The relatively lower use of ITNs in ‘least poor’ households might be a result of the lack of perceived vulnerability, as participants reported the lack of mosquitoes as a disincentive for bed net use. While associations with socio-economic status were heterogeneous across the three sites, these findings mirror reports of socioeconomic differentials in previous studies in sub-Saharan Africa, and most likely reflect the complex pathways that poverty influences malaria prevention practices [36]. However, regardless of the direction of the relationships, there is need for ITN distribution mechanisms and educational interventions that account for socio-economic differentials in ITN uptake and use. Our findings, in conjunction with those of previous studies, also strongly argue for the need to target individuals aged 5–14 years, who continue to emerge as a vulnerable population [7, 37, 38].

There are several limitations in interpreting the findings. First, given the cross-sectional nature of the data, causal associations between malaria knowledge and malaria prevention practices cannot be inferred. Second, the definition of the outcome, bed net use, was based on a question “Did you sleep under a bed net” which may not fully capture the temporal variations in bed net use. Furthermore, as the measure was based on self-report, it may have been subject to recall or social desirability bias. Third, the exposure of interest—knowledge of ITNs as a preventive measure—captures only one aspect of the broader concept of malaria knowledge and only one preventive measure. Fourth, the present study determined individual and household-level factors associated with bed net use; contextual factors such as country specific policies and implementation strategies may further explain bed net use. Furthermore, the primary objective of the broader community based survey was not to assess malaria knowledge, therefore, a limited number of open-ended questions specific to the cause, symptoms and prevention of malaria were selected to minimize response burden. Nevertheless, findings from this study give insights into the level of knowledge and the use of the same standardized questionnaire and indicator definitions allowed the examination of possible variations by study site. Further in-depth studies more appropriate methods such as knowledge, attitude, and practice (KAP) surveys and focus group discussions (FGDs) are warranted [39].

## Conclusions

Knowledge of malaria in a large sample of residents in Zambia and Zimbabwe was good, and knowledge of the protective efficacy of ITNs was associated with bed net use. Other associations identified attest to the need for multipronged and context specific approaches to malaria prevention that simultaneously address social, cultural, and structural factors that drive malaria transmission. The considerably lower likelihood of bed net use in children 5–14 years was concerning. Promoting access to ITNs and malaria messaging for school age children should be considered an essential component of broader strategies to control and eliminate malaria in southern Africa and globally.

## Abbreviations

ACT: artemisinin-based combination therapy
aOR: adjusted odds ratio
CI: confidence interval
GEE: generalized estimated equations
IRS: indoor residual spraying
ICEMR: International Centers for Excellence in Malaria Research
ITN: insecticide-treated mosquito net
LLIN: long-lasting insecticide-treated mosquito net
RDT: rapid diagnostic test
